# Pathological and non-pathological hikikomori: social media use, digital engagement, and therapeutic implications

**DOI:** 10.3389/fpsyt.2025.1596504

**Published:** 2025-08-07

**Authors:** Jeff Gavin, Mark Brosnan, Richard Joiner

**Affiliations:** ^1^ Department of Psychology, University of Bath, Bath, United Kingdom; ^2^ Centre for Applied Autism Research, University of Bath, Bath, United Kingdom

**Keywords:** hikikomori, social media, social isolation, pathological, non-pathological, social withdrawal

## Abstract

**Introduction:**

Hikikomori is traditionally defined as a form of pathological social withdrawal marked by extreme social isolation in one’s home, leading to significant functional impairment or distress. However, shifts in working and study habits since COVID-19 have introduced the concept of ‘non-pathological hikikomori’ to describe individuals who are isolated in their homes but do not experience functional impairment or distress. Hikikomori are frequent users of the internet and social media, which raises interesting questions regarding the relationship between social withdrawal and physical withdrawal. This study examined whether social media use differs by hikikomori status (pathological vs. non-pathological) and phase (early [<3 months], pre- [3–6 months], full [6+ months]).

**Method:**

A cross-sectional study recruited 1,420 self-identified frequent internet users (aged 18-25) via Prolific, who completed a questionnaire on their social media activity (time spent; type of communication), hikikomori status (pathological/non-pathological), and phase (early/pre/full). Of these, 1,235 identified as hikikomori (Mage = 21.5, SD = 2.2; females = 661, males = 572, undisclosed = 2). Within this group, 455 were classified as pathological hikikomori (early = 113, pre = 151, full = 191), while 780 were non-pathological (early = 179, pre = 201, full = 400).

**Results:**

Pathological hikikomori used significantly more social media platforms than non-pathological hikikomori (4.16 vs 3.84: F(1,1224)=20.05, p<.001, ηp²=.016). In terms of phase, full hikikomori (3.82) used fewer social media platforms than early (4.01) and pre (4.13) hikikomori, (F(2,1224)=7.19, p<.001, ηp²=.012: early and pre hikikomori did not differ from each other). The interaction between pathological status and phase was not significant [F(2,1224)=1.28, p=.278, ηp²=.002]. Social media platforms were not used for more time by pathological compared to non-pathological hikikomori, but there were differences in how the social media platforms were used. Regarding communication style, across all phases, pathological hikikomori consistently engaged with others via TikTok and YouTube significantly more than non-pathological hikikomori. Using TikTok and YouTube, Pathological hikikomori sent more messages [F(1,958)=8.77, p=.003, ηp²=.009; F(1,1161)=21.50, p<.001, ηp²=.018; respectively] and received more messages [F(1,958)=13.15, p<.001, ηp²=.014; F(1,1161)=21.37, p<.001, ηp²=.018; respectively], had more targeted messages [F(1,958)=8.49, p=.004, ηp²=.009; F(1,1161)=20.77, p<.001, ηp²=.018; respectively], had more stylised messages [F(1,958)=13.60, p<.001, ηp²=.014; F(1,1161)=24.13, p<.001, ηp²=.020; respectively] and had more broadcast messages [F(1,958)=7.58, p=.006, ηp²=.008; F(1,1161)=13.22, p<.001, ηp²=.011; respectively] than non-pathological hikikomori.

**Conclusion:**

These findings suggest a complex relationship between social media use and social withdrawal. Future research should explore whether communication through YouTube and TikTok is linked to social isolation and whether these platforms could serve as intervention tools to support pathological hikikomori. Importantly, as the sample consisted of self-identified frequent internet users, generalizability to the broader population of hikikomori should be treated with caution.

## 1 Introduction

Hikikomori has traditionally been considered a form of social withdrawal whose essential feature is physical isolation in one’s home for 6 months or longer, resulting in significant functional impairment or distress (1–4). Initially, Hikikomori was considered to be a ‘culture-bound’ syndrome unique to Japan (5), however, hikikomori is now recognized as an international phenomenon, associated with social and cultural shifts brought about by modernization, globalization, and the rise of the Internet (1). Hikikomori has been identified in a wide range of countries, including Australia, Bangladesh, Brazil, Canada, China, France, India, Iran, Italy, Oman, South Korea, Spain, Taiwan, Thailand, Ukraine, the USA (3, 6, 7). A recent meta-analysis based on over 58,000 participants from 19 studies within different counties identified a global prevalence rate for hikikomori of 8.0% (95% CI, 4.9%-12.9%; 8). What is more, this prevalence rate of hikikomori did not differ significantly between regions (East Asia and Western), time periods (pre- and post-COVID-19 pandemic), sex, sample size, or presence versus absence of psychiatric disorders (8).

A key feature of Hikikomori is that they are most likely to be frequent internet users (9–13). During COVID-19 restrictions, Gavin and Brosnan (14) conducted an international study of 646 young people (aged 16-24). Consistent with the research above, COVID-19 restrictions led to a significant increase in risk of hikikomori. Counter to previous research, however, an increase in social media use specifically during COVID-19 restrictions reduced hikikomori risk. Thus, whilst there is a strong positive association between frequent internet use and hikikomori generally, during COVID-19 restrictions, increases in social media use decreased hikikomori risk. Recent work following the pandemic has further emphasised the complex role of social media and online engagement in the context of social withdrawal, with some studies identifying both protective and risk factors depending on the types and motivations of online use (15–20). A revision in lifestyle after the COVID-19 pandemic transculturally and ongoing technological advancements – particularly the rise of social media – has paradoxically both exacerbated isolation and provided new forms of social interaction for young adults who confine themselves at home (21). This raises interesting questions concerning the relationship between social media use and hikikomori post-COVID-19 restrictions. Recently, Kato et al. (22) highlighted that, post-COVID-19 restrictions, working and studying at home have become commonplace and isolation in one’s home can be considered ‘the new normal’ rather than pathological. Kato et al. propose a novel concept of ‘non-pathological hikikomori’ for individuals who are physically isolated in their homes but do not experience significant functional impairment or distress associated with this isolation. It may be, therefore, that social media use reduces the risk of developing pathological hikikomori specifically.

In addition, several recent studies conducted since the pandemic, suggest that the long-term impacts of COVID-19 may have altered both prevalence rates and psychosocial dynamics of hikikomori (22–24). This is particularly relevant given that the present study was conducted in 2023-2024, at a time when many of the pandemic-related lifestyle adjustments (such as remote learning, remote work, and increased reliance on digital communication) remained widespread.

Young people use multiple social media platforms (25) and different social media platforms are likely to impact on distress associated with isolation differently. There are a number of ways that social media platforms differ. In terms of architecture, some are predominantly text-based (e.g., Twitter/X), some image-based and video based (Instagram and TikTok), whereas others a combination of both (e.g., Facebook; 26). In terms of motivations, Facebook and Twitter/X are used predominantly for social connection, social support and relational maintenance needs (26–29), Instagram for self-documentation, self-promotion and self-expression (30), while TikTok and YouTube are used primarily for entertainment rather than social purposes (29, 31, 32). Moreover, each of these social media can be used actively (e.g., posting photos or videos, commenting, etc.) or passively (e.g., browsing or scrolling through one’s feed). Measures of general social media use cannot take account of these structural and user-led differences. Burke and Kraut (33) make a useful distinction between three types of social media communication: 1) targeted, composed communication, consisting of original text for a specific person (e.g., a wall post or comment); 2) stylized or one click communication, a form of low effort but targeted communication or feedback (e.g., a like); and 3) broadcast communication such as a status update or tweet aimed at wide audience. All of these can be sent or received by the social media user, and each can affect feelings of distress related to isolation differently. Research on Facebook, for example, indicates that receiving targeted communication is linked to reductions in distress whereas receiving stylized or broadcast communication is not (27–29, 34, 35).

A growing body of research suggests that the affordances of digital platforms play a crucial role in shaping the experiences of hikikomori, particularly in terms of their ability to engage with the outside world in ways that feel safe and manageable. Park and Yap (35) explored how technological affordances support the gradual reintegration of hikikomori into society, identifying three levels of affordances: individual, community, and societal. Their study found that online platforms provide hikikomori with opportunities for anonymous storytelling, meta-connectivity through discussion threads and reactions, peer networking, and skill development. These affordances enable hikikomori to regain confidence, manage anxiety, and form digital relationships that may later transition into offline social interactions.

While Park and Yap focused on individuals who had already reached the full hikikomori stage, their framework of affordances is relevant to earlier hikikomori phases as well. Kato et al. (22) propose there are three key phases for the development of hikikomori. Less than three months of not leaving the home is termed ‘early-hikikomori’, 3–6 months is termed ‘pre-hikikomori’ and 6+ months is termed ‘full-hikikomori’ (whether pathological or non-pathological). The early- (<3 months) and pre- (3–6 months) hikikomori stages involve increasing social withdrawal, but also potential points of intervention. Social media, with its distinct affordances, may play a role in either accelerating withdrawal or mitigating it by maintaining low-pressure social connections. Affordances such as passive engagement (e.g., scrolling through feeds, watching videos) might reinforce withdrawal by enabling social consumption without interaction, while active engagement (e.g., commenting, messaging, or posting) could help sustain social confidence. Importantly, different platforms provide varying affordances; some fostering connection, others reinforcing isolation. By examining how social media is used across the three hikikomori phases, this study aims to clarify whether certain affordances contribute to the prevention or deepening of social withdrawal.

The current study sought to identify whether the use of social media by young people with pathological hikikomori differed from non-pathological hikikomori, and whether this differed by phase (early-/pre-/full-) of hikikomori. The potential relevance of the phases is highlighted by Kubo et al. (24) who recently found a particularly strong tendency towards gaming disorder in those with early-hikikomori specifically. Therefore, we compare pathological and non-pathological hikikomori across these three phases. Operationally, leaving the house 4+ days per week excludes people from being hikikomori (pathological or non-pathological) and this non-hikikomori group are not considered in this study (see Method section).

## 2 Method

### 2.1 Participants

1420 participants were recruited in the USA through an online database (Prolific) and were paid $5. Inclusion criteria were being aged 18 to 25, a native English speaker and being a frequent internet user (reported using the internet frequently or for extended periods each day). Of these 185 were identified as non-hikikomori (see 2.2 below) and were excluded from the analysis, leaving 1235 hikikomori participants (87% of those who accessed the survey, see Method section). Ages ranged from 18 to 25 with a mean of 21.5 years (SD=2.2). The survey included questions assessing participants’ hikikomori status, frequency and type of social media use across platforms (TikTok, YouTube, Facebook, Instagram, Twitter/X), and demographic information. No other psychometric or clinical measures were administered. The demographic variables of the participants are in **Table 1**.

**Table 1:** Demographic information.

Demographic	N (%)
Gender	
Females	661 (53.5)
Males	572 (46.3)
Self-identify	2 (0.2)
Ethnicity	
White	643 (52.1)
Black (African/Afro-Caribbean/other)	262 (21.2)
Hispanic	177 (14.3)
Asian (Bangladeshi/Indian/Pakistani/Chinese/Other)	59 (4.8)
Mixed	56 (4.5)
Other	38 (3.1)
Living circumstances	
Living with family	979 (79.3)
Living alone	163 (13.2)
Living with friends/acquaintances	88 (7.1)
Living with strangers	(0.4)

### 2.2 Methodology

A cross-sectional online survey was employed. Initially demographic variables of gender, age, ethnicity and living circumstances were requested (see **Table 1**). Three questions were then used to characterize participants’ status as either: non-hikikomori; pathological-hikikomori or non-pathological-hikikomori, and phase as early-, pre-, or full-hikikomori:

1) Thinking back over the past week, how often did you leave your home (days per week)?

Rarely (1 day/week or less) – Hikikomori response (pathological or non-pathological)
Occasionally (2–3 days/week) – Hikikomori response (pathological or non-pathological)
Frequently (4+ days/week) – Non-hikikomori response (excluded from analysis)

2) How long has this been usual or typical for leaving your home (in months)?

<3 months – Early-hikikomori response
3 to <6 months – Pre-hikikomori response
6+ months – Full-hikikomori response

It is worth noting that the ‘early’ phase (<3 months) includes a range of durations of social withdrawal, from as little as one week to three months, which may capture both transient and more established patterns of behaviour.

3) During this period, how socially isolated did you feel? (please click one option)

Not at all – Non-pathological hikikomori response
Mildly – Non-pathological hikikomori response
Moderately – Pathological hikikomori response
Severely – Pathological hikikomori response

The criteria and possible responses come from Kato et al. (1, 2, 22). Six categories of hikikomori were formed using these criteria: Pathological status (yes/no) x phase (early/pre/full). Although direct measures of functional impairment and distress were not collected, prior work by Fong et al. (36) has shown that perceived social isolation is highly correlated with both functional impairment and distress within hikikomori-like populations. As such, we treated perceived social isolation as an operational marker for distinguishing between pathological and non-pathological hikikomori status.

#### 2.2.1 Social media use

Use of five social media was assessed (Facebook, Instagram, Twitter/X, YouTube, TikTok). These were selected as they have been identified as popular social media platforms for young people aged 18-24 (25). For each platform, participants identified how long they had been active on the platform in the past week on an average day [from: Never (0); <10 mins (1); 10–30 minutes (2); 31–60 minutes (3); 1–2 hours (4); 2–3 hours (5); 3+ hours (6)].

For each social media that had been used, participants were then asked to identify how the social media had been used on an average day, in the past week. Participants were asked to identify if they had sent or received communications, and whether these communications were targeted, stylized or broadcast (35). Participants rated each of the six categories (sent/received x targeted/stylized/broadcast), from Never (0); 1 to 3 times (1); 4 to 8 times (2); 9 to 15 times (3); More than 15 times (4).

### 2.3 Analysis

Data met assumptions for analyses of variance, with all dependent variables having acceptable skewness and kurtosis between -1 and 1, as well as acceptable homogeneity of variance (Levene’s Test, p>.01). A 2x3 univariate ANOVA was conducted to identify any significant usage differences for those who used each social media platform between hikikomori status (pathological/non-pathological) and phase (early-/pre-/full-). As previous research has identified sex and age differences in social media usage, these variables were controlled for as covariates (2 people who self-identified were removed from analyses by sex as the numbers for this group were so small). As there were five measures of social media use under investigation, a Bonferroni correction was applied, α = .05/5=.01. Partial eta-squared (ηp²) measures are reported for the effect size. Cohen’s benchmarks cover a small effect (ηp² < 0.01), a medium effect (0.01 < ηp² < 0.06), and a large effect (ηp² > 0.06).

### 2.4 Ethics

Ethical approval was received form the Psychology Research Ethics Committee at the University of Bath. All participants provided informed consent to participate.

## 3 Results

**Table 2** highlights the number of pathological and non-pathological hikikomori by phase (early-/pre-/full-). The percentages in **Table 2** refer to the proportion of each group relative to the analysed sample. Most participants were non-pathological (63%) and almost half of the participants were full hikikomori (48%). There was no age differences (both mean=21.5, sd=2.16: t(1232)=.125, p=.900), no differences in the proportion of males and females [Chi^2^(1) = 1.60, p=.209], no differences in ethnicity [Chi^2^(4) = 5.01, p=.286] and no differences in living circumstances [Chi^2^(3) = 1.82, p=.610] between the pathological and non-pathological hikikomori groups. In terms of phase, almost half (48%) of participants were full hikikomori. Once again, there was no significant age differences [means=21.4-21.7, sd=2.1-2.2: F(2,1231)=2.21, p=.110], and no differences in the proportion of males and females [Chi^2^(2) = 0.05, p=.977] across the phases of hikikomori. There was a trend for differences in living circumstances [Chi^2^(6) = 15.92, p=.014] as most hikikomori lived with their families for all three phases of hikikomori, with the exception of those who lived alone. When considering those who lived alone, hikikomori were evenly distributed across all three phases. Finally, there was a significant difference for ethnicity [Chi^2^(8) = 37.23, p<.001] as there was a larger proportion of each ethnicity in the full hikikomori phase (around 50%) except for those reporting a Black identity, which were equally distributed across all three phases of hikikomori.

**Table 2:** Number of hikikomori (status x phase).

Pathological			Non-pathological		
Early	Pre	Full	Early	Pre	Full
113 (9%)	151 (12%)	191 (16%)	179 (15%)	201 (16%)	400 (32%)

### 3.1 Number of social media platforms and time spent on social media platforms

Initially the number of social media platforms used was examined. **Table 3** highlights the number (and percentage) of users for each social media platform. In terms of status, pathological hikikomori used significantly more of these social media platforms than non-pathological hikikomori (4.16 vs 3.84: F(1,1224)=20.05, p<.001, ηp²=.016). In terms of phase, full hikikomori (3.82) used fewer of these social media platforms than early (4.01) and pre (4.13) hikikomori, (F(2,1224)=7.19, p<.001, ηp²=.012: early and pre hikikomori did not differ from each other). The interaction between hikikomori status and phase was not significant (F(2,1224)=1.28, p=.278, ηp²=.002).

**Table 3:** Number (and %) accessing different social media platforms.

Social media platform	Pathological			Non-pathological		
	Early	Pre	Full	Early	Pre	Full
Facebook	71 (63%)	123 (82%)	145 (76%)	137 (76%)	142 (71%)	282 (71%)
Instagram	103 (91%)	144 (95%)	172 (90%)	155 (87%)	181 (90%)	342 (86%)
TikTok	97 (86%)	131 (87%)	152 (80%)	147 (82%)	163 (81%)	277 (69%)
Twitter/X	71 (63%)	110 (73%)	123 (64%)	99 (55%)	122 (61%)	210 (53%)
YouTube	105 (93%)	147 (97%)	181 (95%)	170 (95%)	193 (96%)	375 (94%)

**Table 4** highlights how frequently each of these social media platforms was used, for those who indicated that they used the social media platform (i.e. if the platform was never used by a participant, their data was not included). ANOVA analysis identified that there were no significant difference in the amount of time these social media platforms were used between hikikomori status, phase, or the interaction between hikikomori status and phase. All comparisons were not significant p>.01, all ηp²<.01.

**Table 4:** Number (and %) time spent using each social media platform.

Social media platform	Less than 10 minutes (1)	10–30 minutes (2)	31–60 minutes (3)	1-2 hours (4)	2-3 hours (5)	Over 3 hours (6)
Facebook (N=916)	235 (26%)	215 (24%)	127 (14%)	117 (13%)	82 (9%)	140 (15%)
Instagram (N=1097)	168 (15%)	224 (20%)	205 (19%)	226 (21%)	130 (12%)	144 (13%)
TikTok (N=967)	87 (9%)	141 (15%)	149 (15%)	187 (19%)	172 (18%)	231 (24%)
Twitter/X (N=735)	190 (26%)	168 (23%)	134 (18%)	102 (14%)	71 (10%)	70 (10%)
YouTube (N=1171)	128 (11%)	136 (12%)	197 (17%)	226 (19%)	225 (19%)	259 (22%)

### 3.2 Use of social media platforms

Finally, how each social media was used was analysed. The five dependent variables for each social media platform were number of messages sent, number of messages received, number of targeted messages, number of stylised messages and number of broadcast messages. Taken together, there were three overarching patterns. Firstly, for both TikTok and YouTube, there were consistent differences between pathological and non-pathological hikikomori for all dependent variables. Pathological hikikomori sent more messages [F(1,958)=8.77, p=.003, ηp²=.009; F(1,1161)=21.50, p<.001, ηp²=.018; respectively] and received more messages (F(1,958)=13.15, p<.001, ηp²=.014; F(1,1161)=21.37, p<.001, ηp²=.018; respectively), had more targeted messages [F(1,958)=8.49, p=.004, ηp²=.009; F(1,1161)=20.77, p<.001, ηp²=.018; respectively], had more stylised messages [F(1,958)=13.60, p<.001, ηp²=.014; F(1,1161)=24.13, p<.001, ηp²=.020; respectively] and had more broadcast messages [F(1,958)=7.58, p=.006, ηp²=.008; F(1,1161)=13.22, p<.001, ηp²=.011; respectively] than non-pathological hikikomori.

There were no differences in messaging between the different phases of hikikomori for TikTok and YouTube (all p>.01, ηp²<.01), with the exception for YouTube only for sent messages [F(1,1161)=4.82, p=.008, ηp²=.008], targeted messages [F(1,1161)=7.85, p<.001, ηp²=.013] and stylised messages [F(1,1161)=6.96, p<.001, ηp²=.012]. For all three variables full hikikomori were significantly lower than early- and pre- hikikomori (who were very similar to each other). There were no significant interactions between hikikomori status and phase (all p>.01, all ηp²<.01).

Secondly, for Facebook and Instagram, there were specific significant differences between pathological and non-pathological hikikomori for some, not all, dependent variables. For Facebook there were significant differences for all the variables except stylised messages whereas for Instagram, broadcasting messages was the only significant difference. For Facebook and not Instagram, pathological hikikomori sent significantly more messages [F(1,907)=7.05, p=.008, ηp²=.008; F(1,1087)=6.50, p=.011, ηp²=.006; respectively], received more messages [F(1,907)=6.63, p=.01, ηp²=.007; F(1,1087)=3.91, p=.048, ηp²=.004; respectively], and had more targeted messages [F(1,907)=10.93, p<.001, ηp²=.012; F(1,1087)=1.85, p=.175, ηp²=.002; respectively]. Pathological hikikomori also used both Facebook and Instagram to broadcast more than non-pathological hikikomori [F(1,907)=9.45, p=.002, ηp²=.010; F(1,1087)=7.58, p=.006, ηp²=.007; respectively]. Finally, Facebook and Instagram were not used more for stylised messages by pathological compared to non-pathological hikikomori [F(1,907)=2.60, p=.107, ηp²=.003; F(1,1087)=5.75, p=.017, ηp²=.005; respectively].

There were hikikomori phase differences for Facebook for receiving messages [F(2,907)=5.34, p=.005, ηp²=.012] and stylised messages [F(2,907)=5.70, p=.003, ηp²=.012], with full hikikomori receiving less and using less stylized messages than the other two groups (who were similar to each other). There was one hikikomori phase difference for Instagram in targeted messages [F(2,1087)=5.00, p=.007, ηp²=.009], again with full hikikomori having less targeted messages than the other two groups (who were similar to each other). All other comparisons were not significant, p>.01, all ηp²<.01. There were no significant interactions between hikikomori status and phase (all p>.01, all ηp²<.01).

Thirdly, for Twitter/X, there were no significant differences in sending and receiving messages or in targeted, stylised and broadcast messages for hikikomori status or phase, or the interaction of the two (all p>.01; all ηp²<.01).

## 4 Discussion

### 4.1 Key findings

This is the first study to systematically examine social media use among early-, pre-, and full-hikikomori across both pathological and non-pathological groups. A key finding is that non-pathological hikikomori (63%) outnumbered pathological (37%) cases. That is, many individuals met the criteria for hikikomori (remaining at home most days of the week) but did not report experiencing significant distress. This aligns with research from Japan (22), which suggests that post-COVID-19, working or studying from home has become normalized, potentially contributing to the rise of non-pathological hikikomori. 87% of our initial sample reported remaining at home 4+ days per week, consistent with hikikomori criteria. Crucially, this reflects our sampling of frequent internet users and is not a population estimate of hikikomori (pathological and non-pathological). Additionally, time spent on these social media platforms generally declined as individuals progressed from pre-hikikomori to full-hikikomori, regardless of whether they were classified as pathological or non-pathological. When exploring the use of social media, pathological hikikomori did not use these social media platforms for a different amount of time compared to non-pathological hikikomori, but they did use social media differently. Our findings indicate that pathological hikikomori use a broader range of these five social media platforms than their non-pathological counterparts, with significantly greater engagement on TikTok and YouTube for communication with others.

### 4.2 Social media use among pathological hikikomori

When examining those who used each social media platform, the amount of time spent was comparable between pathological and non-pathological hikikomori. Importantly, there were clear differences in *how* social media platforms were used (mostly with medium effect sizes). Specifically, pathological hikikomori consistently reported using TikTok and YouTube significantly more for all types of communication compared to non-pathological hikikomori. While these platforms are generally associated with entertainment rather than social interaction (29, 31, 32), pathological hikikomori, despite experiencing social isolation, appear to rely on them for communication to a greater extent than non-pathological hikikomori. One possibility is that TikTok and YouTube contribute to social displacement, whereby time spent engaging with digital entertainment reduces time available for face-to-face interactions, potentially worsening withdrawal (15). While this effect has been demonstrated with television and early Internet use (16, 37), there is limited causal evidence that social media use directly displaces offline interaction (15). However, given that TikTok and YouTube function similarly to television, albeit with integrated communicative features, further research is needed to explore whether these affordances could be leveraged to support social reintegration. This is pertinent as our analyses controlled for sex and age, and since the demographic factors (e.g., sex, age, ethnicity, living circumstances) did not differentiate pathological from non-pathological hikikomori, the distinction in social media usage between pathological and non-pathological hikikomori is notable.

Although platforms like TikTok and YouTube are often classified as ‘passive’ media environments, our findings show that pathological hikikomori are actively using these platforms for communication (sending messages, receiving messages, and engaging with targeted and stylised content). In this sense, while the platforms themselves may be passive environments, the nature of their engagement is active in the context of this group.

A useful framework for understanding these differences in how social media platforms can impact on offline interaction comes from Park and Yap’s (35) research on technology affordances in hikikomori reintegration. They identify three levels of affordances that can facilitate a return to social participation: individual, community, and societal. At the individual level, affordances such as anonymous storytelling and meta-connectivity (e.g., discussion threads, likes, and comments) provide low-risk engagement opportunities. At the community level, features that support peer networking, such as private messaging and structured online groups, help strengthen virtual relationships, which may eventually evolve into offline connections. Applying this framework, non-pathological as well as early- and pre- hikikomori may benefit from platforms that foster these types of gradual engagement, such as discussion forums, gaming communities, or private group chats. These affordances help sustain a sense of connection while increasing comfort with social interaction. In contrast, pathological hikikomori appear to be engaging primarily in passive content consumption. Their preference for TikTok and YouTube suggests a reliance on digital spaces that afford entertainment rather than reciprocal interaction, potentially reinforcing withdrawal rather than supporting reintegration.

This pattern of passive engagement is concerning. Research consistently links passive social media use (e.g., scrolling through content without interacting) to negative mental health outcomes, including increased social anxiety (17–20, 38, 39). Experimental studies suggest a causal link, with heightened social media use contributing to greater social anxiety (40). Given that social anxiety frequently co-occurs with hikikomori (41), the high levels of passive engagement among pathological hikikomori may contribute to worsening psychological distress.

Interestingly, some pathological hikikomori may attempt to be active on TikTok and YouTube but struggle with the communicative norms of these platforms. This suggests that their efforts at social interaction may be unsuccessful or misaligned with how these platforms are typically used. For non-pathological as well as early- and pre- hikikomori, encouraging engagement with affordances that support structured, reciprocal interactions, such as moderated online groups, guided content creation, or skill-building platforms, may help maintain social connection while reducing reliance on passive consumption. For pathological hikikomori, interventions may need to focus on disrupting entrenched patterns of passive engagement and guiding individuals toward digital spaces that promote more active participation. Platforms such as Facebook or Instagram could enable gradual steps toward meaningful interaction, potentially helping to counteract the reinforcing cycle of isolation, especially given that these platforms were also used by pathological hikikomori in the present study. Whilst not always reaching statistical significance, pathological hikikomori often tended towards using these social media platforms more than non-pathological hikikomori.

### 4.3 Phase-related differences and implications for early intervention

Although there were fewer consistent differences across the hikikomori phases, we did find evidence that individuals in the full-hikikomori stage (6+ months) engaged with fewer social media platforms overall, and reported less frequency of some types of communication (compared to early-/pre-hikikomori). Although longer−term hikikomori used fewer platforms overall, this does not necessarily imply reduced online communication. Instead, it suggests that for those in later hikikomori phases, social media may remain a conduit for active interaction, albeit within a narrower range of platforms. In this sense, it is how platforms are used, rather than the quantity, that may maintain isolation.

This suggests that prolonged social withdrawal may eventually lead to disengagement from online communication as well, reinforcing isolation. This is an important consideration for early intervention. Originally, hikikomori was only classified as a condition after six months of withdrawal, but identifying individuals earlier may allow for more effective support (22, 24). Japan’s long-term hikikomori cases highlight the risks of prolonged social withdrawal, with 50% of cases persisting for over seven years (4). This has contributed to the ‘8050 problem’ where middle-aged hikikomori rely on elderly parents for support, raising concerns about long-term well-being and care (42). Early intervention is therefore crucial to prevent similar long-term cases in other countries where hikikomori is an emerging phenomenon. There were no significant status by phase interactions in the present study, suggesting that any platform-specific interventions may be comparably appropriate across early-/pre-/full-hikikomori phases.

Interestingly, we observed no significant interaction between hikikomori status and phase, suggesting that the effects of pathology status and progression through hikikomori phases on social media use operate largely independently. This may imply that the mechanisms underlying social media engagement in hikikomori evolve similarly across both pathological and non-pathological groups, regardless of phase.

### 4.4 Conclusions: Towards platform-specific interventions

This study is the first to show that social media use differentiates pathological from non-pathological hikikomori. Our findings challenge the idea that social media necessarily reduces hikikomori risk, as suggested by Gavin and Brosnan (14) during COVID-19 restrictions. Instead, post-pandemic, pathological hikikomori engage with social media in ways that may reinforce rather than mitigate their isolation, particularly through TikTok and YouTube.

While our study did not identify demographic factors distinguishing the two groups, this may reflect the characteristics of our sample (e.g., frequent internet users), which could limit generalizability. Additionally, as a cross-sectional study, we cannot establish causality between social media use and hikikomori severity. Longitudinal research is needed to track social media engagement over time and its potential role in hikikomori progression.

We acknowledge the limitation that this approach does not capture the full breadth of the hikikomori definition (1), which includes distress and functional impairment as core characteristics. Future studies would benefit from incorporating direct measures of distress and functional impairment, to enable a more comprehensive classification of hikikomori status. Given the aim of exploring the relationship between hikikomori and social media use, the sample was recruited online and comprised self-selected, frequent internet users. As such, it should be viewed as representative only of this sample and not as representative of the general US youth population or the wider hikikomori population. It is also worth noting that lower engagement across social media platforms may not necessarily imply lower overall online activity. Participants may have engaged more extensively with other digital spaces (e.g., online gaming environments) that were not assessed in this study.

The early stage of hikikomori is defined as ‘within 3 months from the onset’ [ (22); see also (43)], which this study operationalised at reporting the frequency of mostly staying at home for ‘<3 months’. Recent screens for hikikomori have asked ‘During the past one month, about how many days a week did you go out…’ [ (22); see also (43)] indicating the early phase of hikikomori is identified when weekly frequency of leaving the home is averaged over the first month from onset (up to three months when pre-hikikomori is identified). This early hikikomori phase may include a heterogeneous group of participants, from those only recently beginning to withdraw (a few weeks) to those with a longer, more established period of withdrawal closer to a few months. This range should be considered when interpreting the findings, and future studies might benefit from narrower or more differentiated early-phase categorizations (such as 1–3 months rather than <3 months).

Overall, our findings highlight the complex role of social media in hikikomori experiences. While online platforms may provide some non-pathological hikikomori with a sense of connectedness, for pathological cases, excessive engagement, particularly in passive forms, may contribute to psychological distress and withdrawal. These findings suggest that social media engagement should be considered in therapeutic approaches for hikikomori. Rather than treating all social media use as inherently harmful or beneficial, interventions should focus on platform-specific affordances.

Given that pathological hikikomori engage in passive social media use more than non-pathological hikikomori, strategies that encourage structured, active participation on platforms designed for interaction may be beneficial. For example, platforms with strong social or community-building elements may be more effective in fostering meaningful connections. Additionally, tailored digital interventions could help hikikomori navigate social media in ways that reduce social anxiety rather than exacerbate it. Future research should explore how different platforms might be harnessed to provide support while minimizing potential harms.

Encouraging social media use that aligns with the affordances hikikomori seek, while also guiding them toward platforms that facilitate active rather than passive engagement, could be a promising avenue for intervention. If pathological hikikomori gravitate toward platforms that afford passive engagement, interventions should focus on redirecting their use toward platforms that support structured, reciprocal communication. For example, instead of passively consuming YouTube or TikTok videos, they could be encouraged to participate in guided content creation, moderated discussions, or structured online support communities. By integrating an understanding of platform affordances into interventions, clinicians and researchers may be able to design more targeted support systems that balance online engagement with the broader goal of social reintegration. Future studies should also consider intra−platform differences in user motivation and engagement, as these factors may play a pivotal role in the effectiveness of interventions. Even within platforms like YouTube or TikTok, motivations for use (active communication versus passive viewing) can vary significantly and may critically shape the role these platforms play in either reinforcing or alleviating hikikomori.

## Data Availability

The raw data supporting the conclusions of this article will be made available by the authors, without undue reservation.

## References

[1] Kato TA Kanba S Teo AR. Hikikomori: Multidimensional understanding, assessment, and future international perspectives. Psychiat Clin Neuros. (2019) 73:427–40., PMID: 31148350 10.1111/pcn.12895

[2] Kato TA Kanba S Teo AR. Defining pathological social withdrawal: Proposed diagnostic criteria for hikikomori. World Psychiat. (2020) 19:116–7. doi: 10.1002/wps.20705, PMID: 31922682 PMC6953582

[3] Tan MP Lee W Kato TA. International experience of hikikomori (prolonged social withdrawal) and its relevance to psychiatric research. BJPsych Int. (2021) 18:34–7. doi: 10.1192/bji.2020.20, PMID: 34287404 PMC8274427

[4] Teo AR. A new form of social withdrawal in Japan: A review of hikikomori. Int J Soc Psychiat. (2010) 56:178–85. doi: 10.1177/0020764008100629, PMID: 19567455 PMC4886853

[5] Teo AR Gaw AC. Hikikomori, a Japanese culture-bound syndrome of social withdrawal? A proposal for DSM-V. J Nerv Ment Dis. (2010) 198:444. doi: 10.1097/NMD.0b013e3181e086b1, PMID: 20531124 PMC4912003

[6] Kato TA Tateno M Shinfuku N Fujisawa D Tea AR Sartorius N. Does the ‘hikikomori’ syndrome of social withdrawal exist outside Japan? A preliminary international investigation. Soc Psych Psych Epid. (2012) 47:1061–75. doi: 10.1007/s00127-011-0411-7, PMID: 21706238 PMC4909153

[7] Teo AR Fetters MD Stufflebeam K Tateno M Balhara Y Choi T. Identification of the hikikomori syndrome of social withdrawal: Psychosocial features and treatment preferences in four countries. Int J Soc Psychiat. (2015) 61:64–72. doi: 10.1177/0020764014535758, PMID: 24869848 PMC5573567

[8] Zhang W Chen MY Feng Y Su Z Cheung T Jackson T. Epidemiology of Hikikomori: A systematic review and meta-analysis of 19 studies. Psychiatry Clin Neurosci. (2025) 79:138–46. doi: 10.1111/pcn.13768, PMID: 40016086

[9] Hamasaki Y Nakayama T Michikoshi S Hikida T. Risk factors for severity of social withdrawal in adolescence: Understanding hikikomori as a spectrum. Eur Psychiat. (2021) 64:S632–3. doi: 10.1192/j.eurpsy.2021.1682

[10] Tateno M Teo AR Ukai W Kanazawa J Kubo H Kato TA. Internet addiction, smartphone addiction, and Hikikomori trait in Japanese young adult: Social isolation and social network. Front Psychiat. (2019) 10:455. doi: 10.3389/fpsyt.2019.00455, PMID: 31354537 PMC6635695

[11] Kato TA Shinfuku N Tateno M. Internet society, internet addiction, and pathological social withdrawal: The chicken and egg dilemma for internet addiction and hikikomori. Curr Opin Psychiat. (2020) 33:264–70. doi: 10.1097/YCO.0000000000000601, PMID: 32101902

[12] Stip E Thibault A Beauchamp-Chatel A Kisely S. Internet addiction, hikikomori syndrome, and the prodromal phase of psychosis. Front Psychiat. (2016) 7:6. doi: 10.3389/fpsyt.2016.00006, PMID: 26973544 PMC4776119

[13] MurisPvan der VeenALuijtenBde BieCMeestersC. On your own: an explorative study on the psychopathological and psychosocial correlates of hikikomori symptoms in Dutch adolescents and young adults. Child Psychiatry Hum Dev. (2025), 1–13. doi: 10.1007/s10578-025-01828-0, PMID: 40100551

[14] Gavin J Brosnan M. The relationship between hikikomori risk and internet use during COVID-19 restrictions. Cyberpsych Behav Soc Network. (2022) 25:189–93. doi: 10.1089/cyber.2021.0171, PMID: 35021891

[15] Hall JA Liu D. Social media use, social displacement, and well-being. Curr Opin Psychol. (2022) 46:101339. doi: 10.1016/j.copsyc.2022.101339, PMID: 35395533

[16] Kraut R Patterson M Lundmark V Kiesler S Mukophadhyay T Scherlis W. Internet paradox: A social technology that reduces social involvement and psychological well-being? Am Psychol. (1998) 53:1017. doi: 10.1037/0003-066X.53.9.1017, PMID: 9841579

[17] Brosnan M Gavin J. The impact of higher levels of autistic traits on risk of hikikomori (pathological social withdrawal) in young adults. PloS One. (2023) 18:e0281833. doi: 10.1371/journal.pone.0281833, PMID: 36809281 PMC9942989

[18] Lai F Wang L Zhang J Shan S Chen J Tian L. Relationship between social media use and social anxiety in college students: mediation effect of communication capacity. Int J Env Res Public Health. (2023) 20:3657. doi: 10.3390/ijerph20043657, PMID: 36834357 PMC9966679

[19] Goddard R Holtzman S. Finding similar others online: predictors of social support outcomes in online communities for multiracial people. Curr Psychol. (2024) 43:5765–78. doi: 10.1007/s12144-023-04764-1

[20] Koh GK Ow Yong JQY Lee ARYB Ong BSY Yau CE Ho CSH. Social media use and its impact on adults’ mental health and well-being: a scoping review. Worldviews Evid Based Nurs. (2024) 21:345–94. doi: 10.1111/wvn.12727, PMID: 38736207

[21] Nagai Y Kartar A Pfaltz M Elkholy H. The paradox of hikikomori through a transcultural lens. BJPsych Int. (2025) 22:22–4. doi: 10.1192/bji.2024.38, PMID: 40290477 PMC12022855

[22] Kato TA Sartorius N Shinfuku N. Shifting the paradigm of social withdrawal: a new era of coexisting pathological and non-pathological hikikomori. Curr Opin Psychiatr. (2024) 37:177–84. doi: 10.1097/YCO.0000000000000929, PMID: 38415743 PMC10990035

[23] Kato TA Suzuki Y Horie K Teo AR Sakamoto S. One month version of Hikikomori Questionnaire-25 (HQ-25M): development and initial validation. Psychiat Clin Neuros. (2023) 77:188–9. doi: 10.1111/pcn.13499, PMID: 36305452

[24] Kubo T Horie K Matsushima T Tateno M Kuroki T Nakao T. Hikikomori and gaming disorder tendency: a case-control online survey for nonworking adults. Psychiat Clin Neuros. (2024) 78:77–8. doi: 10.1111/pcn.13614, PMID: 37904301 PMC11488624

[25] Dixon S. Share of Gen Z users in the United Kingdom engaging with selected social media platforms daily in October 2022(2022). Available online at: https://www.statista.com/statistics/1341903/social-media-daily-usage-uk-gen-z/ (Accessed November 9, 2022).

[26] Masciantonio A Bourguignon D Bouchat P Balty M Rime B. Don’t put all social network sites in one basket: Facebook, Instagram, Twitter, Tik Tok, and their relations with well-being during the COVID-19 pandemic. PloS One. (2021) 16:e0248384. doi: 10.1371/journal.pone.0248384, PMID: 33705462 PMC7951844

[27] Park N Lee S. College students’ motivations for Facebook use and psychological outcomes. J Broadcast Electron Media. (2014) 58:4: 601–620. doi: 10.1080/08838151.2014.966355

[28] Vaterlaus JM Barnett K Roche C Young JA. Snapchat is more personal”: An exploratory study on Snapchat behaviors and young adult interpersonal relationships. Comput Hum Behav. (2016) 62:594–601. doi: 10.1016/j.chb.2016.04.029

[29] Vaterlaus M Winter M. Tik Tok: an exploratory study of young adults’ uses and gratifications. Soc Sci J. (2021) :1–20. doi: 10.1080/03623319.2021.1969882

[30] Sheldon P Bryant K. Instagram: motives for its use and relationship to narcissism and contextual age. Comput Hum Behav. (2016) 58:89–97. doi: 10.1016/j.chb.2015.12.059

[31] Buf DM Ştefăniţă O. Uses and Gratifications of YouTube: a comparative analysis of users and content creators. Rom J Communic Public Rel. (2020) 22:75–89.

[32] Lu X Lu Z Liu C. Exploring Tik Tok use and non-use practices and experiences in China. In: International conference on human-computer interaction. Springer, Cham (2020). p. 57–70.

[33] Burke M Kraut RE. The relationship between Facebook use and well-being depends on communication type and tie strength. J Comput Mediat Commun. (2016) 21:265–81. doi: 10.1111/jcc4.12162

[34] Bossen CB Kottasz R. Uses and gratifications sought by pre-adolescent and adolescent TikTok consumers. Young Consumers. (2020) 21:463–78. doi: 10.1108/YC-07-2020-1186

[35] Park HE Yap SF. Technology affordances and social withdrawal: the rise of hikikomori. Psych Marketing. (2024) 41:1469–88. doi: 10.1002/mar.21991

[36] Fong TC Cheng Q Pai CY Kwan I Wong C Cheung SH. Uncovering sample heterogeneity in gaming and social withdrawal behaviors in adolescent and young adult gamers in Hong Kong. Soc Sci Med. (2023) 321:115774. doi: 10.1016/j.socscimed.2023.115774, PMID: 36796169

[37] Kraut R Kiesler S Boneva B Cummings J Helgeson V Crawford A. Internet paradox revisited. J Soc Issues. (2002) 58:49–74. doi: 10.1111/1540-4560.00248

[38] Erliksson OJ Lindner P Mortberg E. Measuring associations between social anxiety and use of different types of social media using the Swedish social anxiety scale for social media users: a psychometric evaluation and cross-sectional study. Scand J Psych. (2020) 61:819–26. doi: 10.1111/sjop.12673, PMID: 32713014

[39] O’Day EB Heimberg RG. Social media use, social anxiety, and loneliness: a systematic review. Comp Hum Behav Rep. (2021) 3:100070. doi: 10.1016/j.chbr.2021.100070

[40] Yang F Li M Han Y. Whether and how will using social media induce social anxiety? The correlational and causal evidence from Chinese society. Front Psychol. (2023) 14:1217415. doi: 10.3389/fpsyg.2023.1217415, PMID: 37842706 PMC10570417

[41] Alkis Y Kadirhan Z Sat M. Development and validation of social anxiety scale for social media users. Comp Hum Bahav. (2017) 72:296–303. doi: 10.1016/j.chb.2017.03.011

[42] Yoshioka-Maeda K. The ‘8050 issue’ of social withdrawal and poverty in Japan’s super-aged society. J Adv Nurs. (2020) 76:1884–5. doi: 10.1111/jan.14372, PMID: 32242980

[43] Teo AR Horie K Kurahara K Kato TA. The Hikikomori Diagnostic Evaluation (HiDE): a proposal for a structured assessment of pathological social withdrawal. World Psychiat. (2023) 22:478–9. doi: 10.1002/wps.21123, PMID: 37713577 PMC10503919

